# Next-Generation Sequencing for Identifying Unknown Pathogens in Sentinel Immunocompromised Hosts

**DOI:** 10.3201/eid2902.221829

**Published:** 2023-02

**Authors:** Jay A. Fishman

**Affiliations:** Massachusetts General Hospital Transplant Infectious Disease and Compromised Host Program and Harvard Medical School Transplant Center, Boston, Massachusetts, USA.

**Keywords:** next-generation sequencing, antimicrobial resistance, unknown pathogens, immunocompromised patients, sentinel hosts, hepatitis, viruses

The importance of obtaining a specific microbiologic diagnosis to guide antimicrobial therapy takes on greater urgency in persons immunocompromised after organ or stem cell transplantation or chemotherapy, or with other immune deficits (*1*). These patients tolerate invasive infection poorly, sometimes caused by organisms not previously known to be pathogenic. Incompletely treated or persistent infections in such persons serve as substrates for developing antimicrobial resistance and the evolution of viral mutants unresponsive to natural or vaccine-induced immunity (*2*). Specific diagnoses of infectious syndromes, such as febrile illnesses, pneumonitis, hepatitis, meningitis, and gastroenteritis, despite their relative frequency, are often elusive in immunocompromised hosts. Identifying pathogens using traditional culture systems for bacteria and fungi, even when supplemented by use of organism-specific protein or molecular diagnostics, is limited by requiring comparison with known human pathogens stored in datasets. Identifying viral pathogens in the absence of specific nucleic acid tests might require cell-based culture systems, which are labor-intensive, costly, and inefficient.

Clinical microbiology laboratories are increasingly adopting agnostic metagenomic analysis of clinical samples based on next-generation sequencing (NGS) to identify unexpected or novel pathogenic microorganisms (*3*). NGS platforms, which vary in sensitivity and specificity, enable high-throughput parallel sequencing of nucleic acid fragments in blood and tissues without need for specific targets (*3*). Bioinformatics software is used to reassemble sequenced fragments into a microbial genome, an approach that can be applied to all microbial groups if comparator sequences exist, as well as to identify mutations, resistance markers, and virulence factors. 

In this issue, Philippe Pérot et al. report a novel circovirus causing hepatitis in an immunosuppressed heart-lung recipient who had no clear exposures to common sources of infection (*4*). The virus, tentatively called human circovirus type 1 (HCirV-1), was associated with hepatic injury in this case. Identified using NGS and specific primers with the reassembled sequence, HCirV-1 was detected at high levels in blood and by hybridization in ≈2% of hepatocytes examined. Infection resolved after doctors reduced immunosuppression medications.

The source of HCirV-1 remains uncertain, as is common for infections in immunocompromised hosts. Demonstrating the microbial etiology of infection in this case enabled healthcare providers to appropriately manage treatment by reducing immune suppression medication and avoid unnecessary use of antimicrobial therapies. Among the challenges of NGS have been costs (although they have been decreasing), detection of sequences of unknown clinical significance, and gaps in databank records for many viral and other species for comparison with detected sequences, notably among nonhuman species. Thus, new zoonotic exposures or recombinant species may be unrecognized or masked by any of numerous unknown nucleic acids.

The case described by Pérot et al. is unusual because no other contaminating nucleic acids were detected. Related porcine circoviruses (PCV) 1–4 are heterogeneous and thought to be largely species-specific (*5*). Two strains, PCV2 and PCV3, cause liver infection with prolonged viremia, which is exacerbated by immunosuppression in baboon recipients of porcine cardiac xenografts (*6*–*9*). Human circoviruses have been identified in human feces samples, suggesting that human exposure and infections might be ongoing (*10*). In such circumstances, infection might be missed by routine microbiologic techniques and identified by NGS if the sequence data are included in reference databanks.

In settings such as human xenotransplantation from pigs, where concerns about unrecognized donor-derived infections exist, NGS may serve to bridge gaps in standard clinical microbiology (*11*). Comparisons with closely related known viral sequences, including from zoonotic species, will be required to detect unexpected, novel, or emerging pathogens. Routinely applying NGS tools to uncover pathogenesis in cases of clinical syndromes without an etiologic diagnosis may be costly but might enhance recognition of novel pathogens. Interpreting vast amounts of sequence data, including host sequences, remains daunting for clinical application. The availability of baseline serum samples, such as in the case described by Pérot et al., enables identification of novel sequences; prospective research will require such archived specimens for comparison. Although the prospect is exciting, optimal use of NGS in the clinical care of immunocompromised patients remains to be elucidated.
